# Editorial: Neurosensory Alterations From Blast Exposure and Blunt Impact

**DOI:** 10.3389/fneur.2021.674626

**Published:** 2021-04-08

**Authors:** Venkatasivasaisujith Sajja, Joseph B. Long, Catherine C. Tenn

**Affiliations:** ^1^Blast-Induced Neurotrauma Branch, Center for Military Psychiatry and Neuroscience, Walter Reed Army Institute of Research, Silver Spring, MD, United States; ^2^The Geneva Foundation, Tacoma, WA, United States; ^3^Casualty Management Section, Defence Research and Development Canada, Suffield Research Centre, Medicine Hat, AB, Canada

**Keywords:** blast, blunt, neurosensory alterations, clinical, preclinical, computational

Neurosensory perturbations are the most common symptomatology observed following blast and blunt trauma to brain (1–5). Glasgow coma scale (GCS) for civilian injuries and threshold of injury in military personnel heavily rely on the perturbations associated with neurosensory systems to diagnose mild to severe traumatic brain injury. In addition, a growing body of evidence is revealing gender differences in the experiences of men and women following a brain injury (6, 7). The 11 papers presented on the Research Topic of Neurosensory Alterations from Blast Exposure and Blunt Impact highlight the challenges in understanding the disruptive effects on neurosensory systems following traumatic brain injuries experienced by both civilians and military personnel.

## Clinical Studies

Lumba-Brown et al. examined head injury following a blunt trauma in college athletes and documented the prevalence of oculomotor, vestibular, and auditory impairments among concussed athletes, and the effect of sex and sport on symptom prevalence. They found females tended to have a higher incidence of abnormal outcomes on particular concussion assessment tasks as compared- to males. Sergio et al., using fMRI, showed different sex-related patterns of brain activity during rule-based skill performance and suggested that injury to the brain could result in different visuomotor related impairments in males and females. Both these studies indicate that more research is needed to help better understand the relationship between sex, gender and brain injury to improve diagnosis and treatment for both sexes. Modica et al. found that career military breachers tended to report a greater sensitivity to noise or light as well as irritability when compared to non-breachers. In addition, they found a higher level of self-reporting of tinnitus within this group and discussed the challenges encountered when conducting these studies in order to fully characterize long term exposure to blast. Kuchinsky et al. examined service members and veterans with and without a history of TBI and with and without a history of blast exposure to determine the contribution of auditory, cognitive, and posttraumatic stress disorder symptoms—to difficulties understanding speech in complex environments. They determined that TBI status and cognitive function predicted objective speech recognition performance, exposure to blast—subjective hearing complaints, and PTSD symptoms- hearing complaints. Tinnitus was associated with TBI severity and cognitive performance. The authors discuss the limitations of standard audiometric assessments of service members and veterans with a history of blast exposure and/or TBI and why it is important to use more than just pure-tone audiograms when evaluating chronic effects of TBI.

Vartanian et al. used a cross-sectional study design to compare neuropsychological and neurocognitive profiles of breaching instructors and range staff to non-breaching military controls. While there were differences between the groups on several of the tests, the authors concluded that these results should be carefully considered as these disturbances may not be unique to the breaching environment but rather to the broader life experience of the individual. Carr et al. used a retrospect chart review study design to compare a group of soldiers who, by virtue of their occupation, were exposed to multiple blast events over time but were not associated with a diagnosis from acute blast exposure to a group of soldiers who were likely to be deployed, but unlikely to be exposed to any blast. The two groups of soldiers were compared on a wide range of factors such as military occupational specialty (MOS) assignment, diseases, hospitalizations, ambulance encounters, and disability status. Self-reporting of tinnitus appeared to be the one factor that was clearly different between the two groups. These two studies highlight just some of the complexities of studying the impact of occupational blast exposure on health and performance in military personnel. Complementary the these studies is Wang et al., using blood samples from breachers with repeated blast exposures throughout their military careers, identified DNA methylation changes in genes that were linked to symptoms commonly reported by the study participants such as sleep disturbances, tinnitus and headaches. Their results provide some insights on the neurobiological mechanism(s) underlying mild TBI induced by chronic exposure to blast. Thangavelu et al. studied whether TBI-associated blood based biomarkers (such as GFAP, UCHL-1, Nf-L, tau, and amyloid beta peptides), that are commonly linked with exposure to blast overpressure, could also be measured in sniper trainees who were exposed to low levels of overpressure caused by their weapons, specially 0.50 caliber rifles. Data from neurocognitive testing and self-reporting of symptoms were collected to compare with the blood biomarker findings. Serum levels of amyloid beta peptides were found to be elevated in the sniper trainees following low levels of overpressure exposure from rifle fire even with infrequent TBI symptomology.

## Preclinical Studies

Using a rodent model of blast injury, Dickerson et al. examined neuropathological changes within the thalamus and amygdala following repetitive blast events. Blast exposed animals were shown to have a decrease in performance on a test routinely used for measuring motor function and balance and a significant increase in glial activation in the thalamus, but not in the amygdala. The authors discuss the possibility that the thalamic glial changes could be contributing to the vestibulomotor impairment. Seno et al., using a laser-induced shock wave model, have demonstrated that selective serotonin reuptake inhibitors decreased depression like behavior through hippocampal neurogenesis mediated by brain-derived neurotrophic factor and serotonin.

## Computational Studies

Traumatic optic neuropathy refers to optic nerve injury resulting from direct and indirect head and facial trauma. A direct and an indirect traumatic event can affect the optic nerve, causing impairment of vision (8, 9). Li et al. developed a computational head model with a biofidelic orbit which included the entire length of the optic nerve. This model was used to perform simulations to better understand the mode and location of injury to the optic nerve following an impact to the head from different directions using either a rigid or compliant impactor. The simulated data suggested that impacts to the forehead (vs. the side or back of the head) are more likely to cause optic nerve injury. It was proposed that the mechanisms of injury could be due to “the uneven deformation of the optic canal which induces deformation of the optic nerve and the tugging between the brain and optic nerve.” The development of models (animal models or computational models) of indirect traumatic optic neuropathy (ITON) could prove to be beneficial in understanding the mechanisms of damage as well as potential therapies.

## Author Contributions

All authors listed have made a substantial, direct and intellectual contribution to the work, and approved it for publication.

## Conflict of Interest

The authors declare that the research was conducted in the absence of any commercial or financial relationships that could be construed as a potential conflict of interest.

## References

[1] Assi H Moore RD Ellemberg D Hebert S. Sensitivity to sounds in sport-related concussed athletes: a new clinical presentation of hyperacusis. Sci Rep. (2018) 8:9921. 10.1038/s41598-018-28312-1 29967340 PMC6028444

[2] Hoover EC Souza PE Callun FJ. Auditory and cognitive factors associated with speech-in-noise complaints following mild traumatic brain injury. J AMCad Audiol. (2017) 28:325–39. 10.3766/jaaa.16051 28418327 PMC5600820

[3] Hoffer ME. Mild traumatic brain injury: neurosensory effects. Curr Opin Neurol. (2015) 28:74–7. 10.1097/WCO.0000000000000164 25502047

[4] Swan AA Nelson JT Swinger B Jaramillo CA Eapen BC Packer M. Prevalence of hearing loss and tinnitus in Iraq and Afghanistan veterans: a chronic effects of neurotrauma consortium study. Hear Res. (2017) 349:4–12. 10.1016/j.heares.2017.01.013 28153668

[5] Sajja VSSS LaValle C Salib JE Misista AC Ghebremedhin MY Ramos AN. The role of very low level blast overpressure in symptomatology. Front Neurol. (2019) 10:891. 10.3389/fneur.2019.00891 31555194 PMC6722183

[6] Niemeier JP Perrin PB Hocomb MG Rolston CD Artman LK Lu J. Gender differences in awareness and outcomes during acute traumatic brain injury recovery. J Women's Health. (2014) 23:573–80. 10.1089/jwh.2013.4535 24932911 PMC4089016

[7] Gupte R Brooks W Vukas R Pierce J Harris J. Sex differences in traumatic brain injury: what we know and what we should know. J Neurotruama. (2019) 36:3063–91. 10.1089/neu.2018.6171 30794028 PMC6818488

[8] Steinsapir KD Golberg RA. Truamatic optic neuopathy: an evolving understanding. Am J Opthamol. (2011) 151:928–33. 10.1016/j.ajo.2011.02.007 21529765

[9] Singman EL Daphalapurkar N White H Nguyen TD Panghat L Chang J. Indirect traumatic optic neuropathy. Military Med Res. (2016) 3:2. 10.1186/s40779-016-0069-2 PMC4709956 26759722

